# Enhanced numeracy skills following team-based learning in United States pharmacy students: a longitudinal cohort study

**DOI:** 10.3352/jeehp.2022.19.29

**Published:** 2022-10-27

**Authors:** Rob Edwin Carpenter, Leanne Coyne, Dave Silberman, Jody Kyoto Takemoto

**Affiliations:** 1 Soules College of Business, The University of Texas at Tyler, Tyler, TX, USA; 2 College of Pharmacy, West Coast University, Irvine, CA, USA; 3 Program of Administrative Studies, Boston University Metropolitan College, Boston, MA, USA; Hallym University, Korea

**Keywords:** Literacy, Mathematics, Pharmacy students, Critical thinking, United States

## Abstract

**Purpose:**

The literature suggests that the ability to numerate cannot be fully understood without accounting for the social context in which mathematical activity is represented. Team-based learning (TBL) is an andragogical approach with theoretical links to sociocultural and community-of-practice learning. This study aimed to quantitatively explore the impact of TBL instruction on numeracy development in 2 cohorts of pharmacy students and identify the impact of TBL instruction on numeracy development from a social perspective for healthcare education.

**Methods:**

Two cohorts of students were administered the Health Science Reasoning Test-Numeracy (HSRT-N) before beginning pharmacy school. Two years after using TBL as the primary method of instruction, both comprehensive and domain data from the HSRT-N were analyzed.

**Results:**

In total, 163 pharmacy student scores met the inclusion criteria. The students’ numeracy skills measured by HSRT-N improved after 2 years of TBL instruction.

**Conclusion:**

Numeracy was the most significantly improved HSRT-N domain in pharmacy students following two years of TBL instruction. Although a closer examination of numeracy development in TBL is warranted, initial data suggest that TBL instruction may be an adequate proxy for advancing numeracy in a cohort of pharmacy students. TBL may encourage a social practice of mathematics to improve pharmacy students’ ability to numerate critically.

## Introduction

### Background/rationale

There are varying conceptualizations of numeracy in education. In general, numeracy is often described as being literate in mathematics. However, this description does not encompass the social practice of mathematics imbued in our sociality. The literature suggests that numeracy cannot be fully understood without critically accounting for the social context in which mathematical activity is represented. Thus, we borrow from Lerman [1] and Perso [2] and define numeracy as the disposition and capacity to use mathematics as a social practice to function critically in society. Here, we apply this definition to pharmacy education and consider a social practice perspective rather than a perspective isolated solely in mathematical competence. Because numeracy skills are central to pharmacy education and practice, there is an expectation that pharmacy graduates will critically numerate as a core outcome of their pharmacy education and transition numeracy skills to active practice. Promoting numeracy through effective learning systems requires andragogical consideration that stimulates numeracy experiences through communities of practice, and much of this attention has been directed toward active-learning environments. The challenge is to design active learning strategies that develop and sustain positive numeracy habits of mind.

To better understand active-learning strategies that develop numeracy, the related idea of critical thinking as a core aspect of numeracy development should be considered. Numeracy involves the core skill of thinking critically, and Jain and Rogers [3] recently added support for this notion that critical thinking provides the cognitive platform to apply numeracy. Active learning strategies that promote critical thinking have been a focus of educators for some time. In contrast to traditional learning methods—assigning home reading supplemented by lectures and response work—active learning encourages students to learn through collaborative thinking, structured discourse, and other forms of community practice. In the healthcare professions, active-learning environments have promoted critical thinking habits in various settings. Andragogical strategies that foster the development of higher-order critical thinking skills are also frequently found in pharmacy curricula. Accordingly, since numeracy development involves the core skill of thinking critically, there is a need to understand better the relationship between critical thinking and numeracy in active-learning environments. An active learning andragogy with theoretical links to critical thinking is team-based learning (TBL). Although TBL has seen longitudinal success in pharmacy education for actively developing the skill to think critically [4], the pharmacy education literature is void of studies that investigate improved numeracy as the salient quality of critical thinking post-TBL instruction.

### Objectives

This study aimed to determine whether enhanced numeracy skills are a potential consequence of improved critical thinking post-TBL instruction. Additionally, this study would characterize the context of TBL as a social condition that may play a part in improving numeracy in pharmacy students.

## Methods

### Ethics statement

This study was approved by the Institutional Review Board at the University of Texas at Tyler (SUM2015-103). Informed consent was obtained from the participants.

### Study design

This was a retrospective longitudinal quantitative study involving 2 cohorts of pharmacy students, with data collected from each cohort at 2 points in time. It is described according to the STROBE (Strengthening the Reporting of Observational Studies in Epidemiology) statement (https://www.strobe-statement.org/).

### Setting

The study was carried out at the University of Texas at Tyler Ben and Maytee Fisch College of Pharmacy from 2015 to 2018. The 2 cohorts of pharmacy students examined in this study were enrolled in a pharmacy program that utilized TBL tenets for all didactic instruction [5]. Student teams were randomly formed consisting of 5–6 members at the start of each semester, and the teams were changed each semester thereafter. TBL pedagogy encourages a hierarchy of steps that requires students’ advance preparation, readiness assurance, and application exercises to reinforce course concepts. Students’ advance preparation included pre-class reading and learning objectives guided by module goals; materials were delivered in various formats.

Once arriving at class, each student took a 5- to 20-question multiple-choice individual readiness assurance test (iRAT). Then, each team took the same test as a group readiness assurance test (gRAT). This required each team to engage in intra-group feedback and come to a team consensus on the answers. Any discrepancy between the students’ iRAT and the gRAT was discussed openly through intra-group feedback to reinforce the content of the fundamental concepts. Any remaining unclear concepts were then clarified with inter-group feedback through class discussion. Following class discussion and a clear understanding of the concepts, teams spent the remaining time in class engaged in solving challenging real-world problems (application exercises) that further demonstrated the material being taught in an active learning context. Structurally, the application exercises followed the 4S framework (significant problem, same problem, specific choice, and simultaneous report) and often concluded with multiple-choice questions or gallery walk-style activities to reinforce the underlying concepts once again. Accordingly, the TBL method of instruction imbues a social dimension to the classroom that essentially functions to actively amplify a student's social and intellectual experience [6].

### Participants

The target participants were 190 students who were enrolled in a PharmD program at a regional university in East Texas, the University of Texas at Tyler Ben and Maytee Fisch College of Pharmacy. They were administered the Health Science Reasoning Test-Numeracy (HSRT-N) before the commencing year 1 of their pharmacy school fall semesters of 2015 and 2016 and were re-administered the HSRT-N after 2 years of TBL instruction in the fall semesters of 2017 and 2018, respectively. The inclusion criteria were all pharmacy students (n=190) who initially took the HSRT-N upon entry into the 2015 and 2016 cohorts. Exclusion criteria were incomplete HSRT-N data for the longitudinal analysis (e.g., students who dropped out and did not take the second administration of the HSRT-N test). Twenty-seven students were excluded due to incomplete HSRT-N data, leaving 163 qualified participants.

### Variables

The variables included 8 domains: analysis, inference, interpretation, evaluation, explanation, induction, deduction, and numeracy.

### Data sources/measurement

The HSRT-N used in this study is from Insight Assessment (www.insightassessment.com). The HSRT-N is a 38-item multiple-choice test that measures reasoning and decision-making processes (Supplement 1). Response data from this assessment were provided by Insight Assessment. The data included an individual test-taker report for each participant that illustrated scores for each domain tested (i.e., interpretation, analysis, inference, explanation, evaluation, induction, numeracy, and deduction) and a group summary report for each cohort at each period. Although the HSRT-N does not provide individual participant responses for each question item, an aggregate of the overall reasoning for critical thinking and each domain-specific cognitive skill is provided. The assessment is specifically predictive of critical thinking skills in health science students. It has been repeatedly proven to have strong psychometric properties, with reliability coefficients ranging between 0.77 and 0.83 for all graduate, undergraduate, and technical or community college settings and multiple healthcare domains [7,8]. The HSRT-N quantifies critical thinking by providing participants with an overall score and specific domain scores on a scale of 50–100. The score ranges are stratified as superior (89–100), strong (81–88), moderate (72–80), weak (63–71), or not manifested (50–62). The higher the score, the stronger the participant’s critical thinking skills. The data used in this study are a subset of data collected to examine pharmacy students’ critical thinking ability before and after 2 years of TBL instruction [4]. Only numeracy, out of the 8 domains, was selected for this study (Dataset 1).

### Bias

Convenience sampling can produce a bias in which some members of the population are less likely to be included than others. The variables of gender, age, race, and degree level from the 2015 and 2016 cohorts were compared with variable data from a population of pharmacy students from the 2017 and 2018 cohorts (of the same institution) to account for sampling bias. A good variance of the mean determined homogeneity between the populations accounting for sampling bias concerns. However, the variables were not compared to pharmacy student cohorts from other pharmacy institutions.

### Study size

The study size was informed by a convenience sample (n=163) of student pharmacists (Table 1). No study size estimation was done because the target students were all participants.

### Statistical methods

Graphical data and mean scores were determined using Microsoft Excel ver. 16.0 (Microsoft Corp., Redmond, WA, USA). Comparisons of HSRT-N scores before and after TBL instruction were performed using the paired Student t-test. A comparison of HSRT-N scores for students who did and did not improve their scores was performed using the Welch t-test. Data were analyzed using JASP ver. 0.10.2 (The JASP Team, Amsterdam, The Netherlands).

## Results

Data from 163 students enrolled in the fall 2015 and 2016 semesters were included in this research. Statistically significant (P<0.001) increases in overall student HSRT-N scores and percentile rank scores were identified (Table 2). Notably, of the 8 domains tested on the HSRT-N, numeracy was the domain in which students demonstrated the most significant overall mean percent increase (P_i_=0.068) following 2 years of TBL instruction (Table 3). This finding allowed us to consider the social context of TBL instruction as a contributing factor.

## Discussion

### Interpretation

Within the health sciences, nurses, medical students, and physicians often demonstrate a lack of confidence in numeracy and struggle with basic conversions, probability analysis, and risk based on numerical data. Bullen et al. [9] found that while 60.9% of pharmacy students passed a multiple-choice numeracy assessment, only 27.9% passed a free-text answer evaluation. This suggests that while most students may recognize a correct answer from limited options, most pharmacy students may be inadequately prepared to engage in numeracy as a competent skill. Moreover, pharmacy students often lack the numeracy proficiencies necessary for pharmacy practice and the extent of numeracy shortcomings in pharmacy education is likely underestimated [10]. This emphasizes the importance of purposeful instructional design in pharmacy curricula to include opportunities to think critically and develop skills to numerate effectively as a health care provider.

Many sources have acknowledged active learning environments that incorporate communities of practice can be a potent solution for facilitating students’ understanding of complex problems. TBL instruction is an andragogical approach that engages pharmacy students to solve complex problems and prepares them to manage clinical situations with increased confidence. Importantly, TBL has also improved pharmacy students’ ability to think critically [4]. This study extends this notion to numeracy by adopting the recent work by Jain and Rogers [3] that places critical thinking as a core cognitive element for numeracy development. Critical thinking and numeracy are essential characteristics pharmacists use to solve complex problems. The results of our research add support to the critical thinking-numeracy linkage, suggesting that TBL instruction may be a potential learning source to develop numeracy among pharmacy students.

By refocusing critical thinking to include numeracy development through TBL, pharmacy students can experience mathematics through real-world sociocultural experience(s) that enable numeracy participation as ethical and active citizens of their group context. This retrospective review of HSRT-N numeracy data supports the conceptual association of critical thinking and numeracy. It also implores us to consider the social function context of TBL instruction as a potential facilitator of numeracy development. Specifically, numeracy concepts should include communities of practice that reflect how students numerate in the real world and how numeracy is shaped by ongoing sociocultural experiences. We suggest that TBL instruction may achieve this goal by providing students an opportunity to learn numeracy frameworks in a social practice format during the readiness assurance process and application exercises. The iRAT and gRAT provide consistent formative feedback, facilitated measurement of knowledge acquisition, and precise comprehension. Then, application exercises reinforce students critical thinking across a problem set to consider multiple solutions—forming a thinking habit of mind beneficial to all forms of decision-making, including numeracy. Finally, the shared discussions encouraged by TBL instruction permit students to consider peer perspectives and communal norms essential for social group learning [11]. The social practice of TBL instruction provokes decision-making facilitated by critical thinking for problem-solving [12]. Aligned with other studies, our findings demonstrate that TBL instruction can potentially increase pharmacy students’ ability to think critically. Furthermore, an extension of our findings indicates TBL instruction may also be beneficial for forming a thinking habit of mind to improve a student's ability to numerate—considered here as a potential consequence from the social practice view of TBL instruction.

We also must consider that engaging students socially to learn mathematics is critical for improving students’ numeracy knowledge [13]. TBL is effective for introducing social conditions within a community of practice where students relate to team norms at the center of student learning [14]. Carpenter et al. [14] extended student engagement for a better understanding of the subjective construal underlying engagement in TBL and we can apply their findings to numeracy development. Their work demonstrated the social need to cognitively relate is the students’ primary mode of engaging in TBL instruction. This means that students draw support from social trust and communities of practice learning toward task achievement.

Extending this perspective to numeracy knowledge suggests that numeracy development may be shaped by TBL communities of practice that require socially engaged problem-solving in a real-world mathematical manner. Viewing numeracy as a social practice within pharmacy education exploits how students’ numeracy practices have sociocultural contingencies. O’Keeffe and Paige [15] add support to this view by arguing mathematical analysis should focus based on important issues in the students’ lifeworld. A key perspective of TBL instruction is that it emphasizes a student’s’ role in team learning as a social practice grounded in knowledge acquisition from their sociocultural-based lived experience(s). This understanding further highlights the importance of social triggers in any andragogical application of numeracy development. Accordingly, we suggest this study supports the idea that TBL instruction may offer a community of practice experience to sublimate critical thinking towards numeracy development from a social practice view.

### Limitations

This research was subject to a regional limitation because the data were obtained from 1 institution (the University of Texas at Tyler Ben and Maytee Fisch College of Pharmacy); thus, further studies at different institutions using TBL instruction would be beneficial. All instructors were trained in the implementation of TBL; however, not all instructors were certified in TBL.

### Suggestions

A TBL approach to numeracy may provide a rich classroom environment to help develop and apply mathematics in various healthcare education paradigms. The community-of-practice experience of pre-class preparation, readiness assurance, and application exercises can stimulate students to engage and render opportunities to think critically—the cognitive foundation of numeracy. The interpretation of many healthcare diagnostics and related medical decision-making has become more complicated and dependent on mathematically complex solutions. There is a growing need to enhance curricular design and incorporate andragogical approaches that prepare future healthcare professionals to be numerate successfully. Additionally, numeracy is consequential in understanding attitudes and behaviors associated with health-related recommendations. For example, the recent coronavirus disease 2019 (COVID-19) pandemic exposed sociocultural challenges to healthcare practices that could have benefited from greater use of social numeracy. An inability to effectively communicate healthcare recommendations can give rise to misinformation, medical hypervigilance, or reckless behavior.

Numeracy is acutely linked to healthcare decision-making and outcomes. Yet, healthcare practitioners have shown inadequate skills in essential tasks like interpreting and communicating basic patient screening statistics. More of today’s healthcare practices, post-COVID-19 and beyond, demand problem-solving that orients social participation within healthcare solutions. Healthcare professionals must seek to increase numeracy skills and be competent conduits of medical information for the benefit of society, and TBL instruction may offer this opportunity. Our findings suggest that TBL instruction may offer meaningful implications to improve numeracy skills across multiple healthcare delivery paradigms. We encourage further research on this andragogical approach in various healthcare education settings.

### Conclusion

This study demonstrates enhanced numeracy skills of pharmacy students following two years of TBL instruction. The data showed that numeracy skills were the most significantly improved HSRT-N domain. By adopting critical thinking as a core element of numeracy, we aptly centered TBL instruction—including its sociocultural and communities of practice context—important for advancing pharmacy students’ numeracy skills. Although a closer examination of numeracy development in TBL is warranted, the initial data suggest that TBL may be an adequate proxy for advancing numeracy in pharmacy students.

## Figures and Tables

**Table 1.** Demographics of study participants^a)^

Characteristic	Cohort		
	All students (n=163)	2015 (n=69)	2016 (n=94)
Sex			
Female	86 (52.8)	33 (47.8)	53 (56.4)
Male	77 (47.2)	36 (52.2)	41 (43.6)
Race/ethnicity			
American Indian or Alaska Native	1 (0.6)	0	1 (1.1)
Asian	33 (20.2)	14 (20.3)	19 (20.2)
Black or African American	41 (25.2)	13 (18.8)	28 (29.8)
Latino/Hispanic	28 (17.2)	15 (21.7)	13 (13.8)
White	52 (31.9)	23 (33.3)	29 (30.9)
2 or more	5 (3.1)	1 (1.4)	4 (4.3)
Other	3 (1.8)	3 (4.3)	0
Age at study onset (yr)			
18–24	68 (41.7)	42 (60.9)	26 (27.7)
25–29	59 (36.2)	16 (23.2)	43 (45.7)
30–34	16 (9.8)	4 (5.8)	12 (12.8)
35–39	11 (6.7)	3 (4.3)	8 (8.5)
>40	9 (5.5)	4 (5.8)	5 (5.3)
Highest degree possessed			
2 Years	31 (19.0)	9 (13.0)	22 (23.4)
4 Years	78 (47.9)	37 (53.6)	41 (43.6)
Professional degree	7 (4.3)	3 (4.3)	4 (4.3)
Other	47 (28.8)	20 (29.0)	27 (28.7)

Values are presented as number (%).

a) These data were previously published (Silberman D, et al. Curr Pharm Teach Learn 2021;13:116-121 [4]) and have been republished here with permission.

**Table 2.** Mean and percentile pre-test and post-test scores in the HSRT-N (n=163)^a)^

Variable	Pre-test	Post-test	P-value
Overall HSRT-N score	76.4±8.1	80.6±6.9	<0.001
Percentile rank score	31.3±25.1	44.2±26.8	<0.001

Values are presented as mean±standard deviation. HSRT-N, Health Sciences Reasoning Test-Numeracy.

a) These data were previously published (Silberman D, et al. Curr Pharm Teach Learn 2021;13:116-121 [4]) and have been republished here with permission.

**Table 3.** Mean and percentile differences between pre-test and post-test scores overall and in each subdomain of the HSRT-N (n=163)^a)^

Variable	No. of students	HSRT-N score			
		Pre-test	Post-test	Percentile change (P_i_)	P-value
Overall score					<0.001
(+) change^b)^	115	75.0 (55–91)	81.8 (64–95)	9.1	
(-) change^c)^	48	79.8 (62–91)	77.6 (61–91)	-2.7	
All students	163	76.4 (55–91)	80.6 (61–95)	5.5	
Analysis					<0.001
(+) change^b)^	99	74.3 (55–91)	84.5 (64–100)	13.7	
(-) change^c)^	64	81.0 (64–100)	77.5 (64–95)	-4.2	
All students	163	77.0 (55–100)	81.8 (64–100)	6.2	
Inference					<0.001
(+) change^b)^	109	74.5 (56–91)	82.3 (66–97)	10.5	
(-) change^c)^	54	81.0 (63–94)	77.8 (59–91)	-4.0	
All students	163	76.6 (56–94)	80.9 (59–97)	5.6	
Interpretation					<0.001
(+) change^b)^	86	67.2 (50–89)	78.9 (61–94)	17.4	
(-) change^c)^	77	76.2 (56–89)	71.7 (56–89)	-5.9	
All students	163	71.4 (50–89)	75.5 (56–94)	5.8	
Evaluation					0.016
(+) change^b)^	74	65.5 (50–83)	73.6 (56–94)	12.3	
(-) change^c)^	89	69.8 (56–94)	65.5 (56–83)	-6.2	
All students	163	67.6 (50–94)	69.2 (56–94)	2.4	
Explanation					<0.001
(+) change^b)^	95	74.5 (50–91)	85.1 (55–100)	14.2	
(-) change^c)^	68	85.0 (64–95)	80.5 (59–95)	-5.2	
All students	163	78.9 (50–95)	83.2 (55–100)	5.5	
Induction					<0.001
(+) change^b)^	102	77.0 (59–94)	84.7 (65–97)	10.0	
(-) change^c)^	61	83.8 (65–94)	81.0 (62–91)	-3.3	
All students	163	79.6 (59–94)	83.3 (62–97)	4.7	
Deduction					<0.001
(+) change^b)^	105	69.7 (50–91)	79.4 (59–100)	13.9	
(-) change^c)^	58	79.4 (59–94)	75.4 (56–94)	-5.0	
All students	163	73.2 (50–94)	77.9 (56–100)	6.4	
Numeracy					<0.001
(+) change^b)^	95	66.4 (50–88)	77 (58–96)	16.0	
(-) change^c)^	68	73.4 (54–92)	69.8 (54–83)	-4.9	
All students	163	69.3 (50–92)	74 (54–96)	6.8	

Values are presented as mean % (range), unless otherwise stated. HSRT-N, Health Sciences Reasoning Test-Numeracy.

a) These data were previously published (Silberman D, et al. Curr Pharm Teach Learn 2021;13:116-121 [4]) and has been republished here with permission.

b) Students who demonstrated an increase in the mean score between the pre-test and post-test.

c) Students who demonstrated no change or a decrease in the mean score between the pre-test and post-test.

## Footnotes

**Data availability**
Data files are available from Harvard Dataverse: https://doi.org/10.7910/DVN/EDCYIC Dataset 1. Raw response data of the numeracy domain from 163 participants collected from the measurement tool, Health Science Reasoning Test-Numeracy.

## Supplementary materials

Supplementary files are available from Harvard Dataverse: https://doi.org/10.7910/DVN/EDCYIC

Supplement 1. Numeracy test item concept of the measurement tool, Health Science Reasoning Test-Numeracy. The complete form of the measurement tool cannot be disclosed due to its commercial status.

Supplement 2. Audio recording of the abstract.
