# Supplementary material for: Enhanced numeracy skills following team-based learning in United States pharmacy students: a longitudinal cohort study
Source: J Educ Eval Health Prof. 2022 Oct 27;19:29. doi: 10.3352/jeehp.2022.19.29 (PMC9811131; doi:10.3352/jeehp.2022.19.29)
Supplement: Supplementary file 2 — Supplement 1. Numeracy test item concept of the measurement tool, Health Science Reasoning Test-Numeracy. The complete form of the measurement tool cannot be disclosed due to its commercial status. [file jeehp-19-29-suppl.docx]

**Supplemental 1.**

The complete form of the **Health Science Reasoning Test (HSRT)** measurement tool cannot be disclosed due to commercial purpose.

**The following information was taken directly from:** <https://www.insightassessment.com/product/hsrt> **regarding the** core concept of each HSRT item domain.

**HSRT score metrics:** OVERALL Reasoning Skills (holistic score for critical thinking skills), Analysis, Interpretation, Evaluation, Explanation, Inference, Deduction, Induction and Numeracy (eight cognitive skill scores to focus future development and training). **Items are drawn from a scientifically developed and tested item pool.**

**OVERALL Reasoning Skills:** The Reasoning Skills OVERALL score describes overall strength in using reasoning to form reflective judgments about what to believe or what to do. To score well overall, the test taker must excel in the sustained, focused, and integrated application of core thinking skills measured on this test, including analysis, interpretation, inference, evaluation, explanation, induction, and deduction. The Overall score predicts the capacity for success in educational or workplace settings which demand reasoned decision making and thoughtful problem solving.

**Analysis:** Analytical skills are used to identify assumptions, reasons, themes, and the evidence used in making arguments or offering explanations. Analytical skills enable us to consider all the key elements in any given situation, and to determine how those elements relate to one another. People with strong analytical skills notice important patterns and details.  People use analysis to gather the most relevant information from spoken language, documents, signs, charts, graphs, and diagrams.

**Interpretation:** Interpretation is the process of discovering, determining, or assigning meaning.  Interpretation skills can be applied to anything, e.g. written messages, charts, diagrams, maps, graphs, memes, and verbal and non-verbal exchanges. People apply their interpretive skills to behaviors, events, and social interactions when deciding what they think something means in a given context.

**Evaluation:** Evaluative reasoning skills enable us to assess the credibility of sources of information and the claims they make.  We use these skills to determine the strength or weakness of arguments.  Applying evaluation skills we can judge the quality of analyses, interpretations, explanations, inferences, options, opinions, beliefs, ideas, proposals, and decisions.  Strong explanation skills can support high-quality evaluation by providing the evidence, reasons, methods, criteria, or assumptions behind the claims made and the conclusions reached.

**Inference:** Inference skills enable us to draw conclusions from reasons, evidence, observations, experiences, or our values and beliefs.  Using Inference, we can predict the most likely consequences of the options we may be considering. Inference enables us to see the logical consequences of the assumptions we may be making. Sound inferences rely on accurate information. People with strong inference skills draw logical or highly reliable conclusions using all forms of analogical, probabilistic, empirical, and mathematical reasoning.

**Explanation:** Explanation is the process of justifying what we have decided to do or what we have decided to believe.  People with strong explanation skills provide the evidence, methods, and considerations they actually relied on when making their judgment. Explanations can include our assumptions, reasons, values, and beliefs. Strong explanations enable others to understand and to evaluate our decisions.

**Induction:** Inductive reasoning relies on estimating likely outcomes. Decision making in contexts of uncertainty relies on inductive reasoning. Inductive decisions can be based on analogies, case studies, prior experience, statistical analyses, simulations, hypotheticals, trusted testimony, and the patterns we may recognize in a set of events, experiences, symptoms or behaviors. Inductive reasoning always leaves open the possibility, however remote, that a highly probable conclusion might be mistaken. Although it does not yield certainty, inductive reasoning can provide a solid basis for confidence in our conclusions and a reasonable basis for action.

**Deduction:** Deductive reasoning is rigorously logical and clear cut. Deductive skills are used whenever we determine the precise logical consequences of a given set of rules, conditions, beliefs, values, policies, principles, procedures, or terminology. Deductive reasoning is deciding what to believe or what to do in precisely defined contexts that rely on strict rules and logic.  Deductive validity results in a conclusion which absolutely cannot be false, if the assumptions or premises from which we started all are true. Deductive validity leaves no room for uncertainty. That is, unless we decide to change the very meanings of our words or the grammar of our language.

**Numeracy:** Numeracy refers to the ability to make judgments based on quantitative information in a variety of contexts. People with strong numeracy can describe how quantitative information is gathered, manipulated, and represented textually, verbally, and visually in graphs, charts, tables and diagrams. Numeracy requires all the core critical thinking skills. Numeracy includes being thoughtfully reflective while interpreting the meaning of information expressed in charts, graphs, or text formats, analyzing those elements, drawing accurate inferences from that information, and explaining and evaluating how those conclusions were reached.
